# Association of nocturia of self-report with estimated glomerular filtration rate: a cross-sectional study from the NHANES 2005–2018

**DOI:** 10.1038/s41598-023-39448-0

**Published:** 2023-08-25

**Authors:** Jianling Song, Ben Ke, Xiangdong Fang

**Affiliations:** Department of Nephrology, The Second Affiliated Hospital of Nanchang University, No. 1, Minde Road, Nanchang, 330006 Jiangxi People’s Republic of China

**Keywords:** Diseases, Nephrology

## Abstract

Nocturia is a manifestation of systemic diseases, in which chronic kidney disease (CKD) is an independent predictor of nocturia due to its osmotic diuretic mechanism. However, to our knowledge, previous studies have not examined the association between nocturia and estimated glomerular filtration rate (eGFR). The purpose of this study was to assess the association between nocturia exposure and eGFR in the general US population. This study presents a cross-sectional analysis of the general US population enrolled in the National Health and Nutrition Examination Survey (NHANES) from 2005 to 2018. To account for potential confounding factors, linear regression analysis was conducted to investigate the association between nocturia and eGFR. Stratified analyses and interaction tests were employed to examine the variables of interest. Additionally, sensitivity analyses were conducted across diverse populations. A total of 12,265 individuals were included in the study. After controlling for confounding factors, the results of the linear regression analysis indicated that a single increase in nocturnal voiding frequency was associated with a decrease in eGFR by 2.0 mL/min/1.73 m^2^. In comparison to individuals with a nocturnal urinary frequency of 0, those who voided 1, 2, 3, 4, and ≥ 5 times at night experienced a decrease in eGFR by 3.1, 5.4, 6.4, 8.6 and 4.0 mL/min/1.73 m^2^, respectively. Nocturia was found to be associated with a decreased eGFR of 4 mL/min/1.73 m^2^ when compared to individuals without nocturia. The sensitivity analysis yielded consistent findings regarding the association between nocturia and eGFR in both CKD and non-CKD populations, as well as in hypertensive and non-hypertensive populations. Nevertheless, inconsistent conclusions were observed across various prognostic risk populations within the CKD context. The presence of nocturia and heightened frequency of nocturnal urination have been found to be associated with a decline in eGFR.

## Introduction

Nocturia, as per the definition provided by the International Continence Society (ICS), refers to the frequency of urination episodes experienced during the main sleep period. It is essential that after the initial awakening for urination, each subsequent urination episode is accompanied by either sleep or the intention to sleep^1^. Nocturia can arise from various etiologies, such as diminished bladder capacity, sleep disorders, and nocturnal polyuria^2–4^. The prevalence of nocturia among adults is notably high, but it tends to be underestimated by both patients and physicians^5^. The implications of nocturia can be severe, encompassing insomnia, debilitation, urinary incontinence, and falls^6^. Moreover, heightened severity of nocturia is linked to a decline in quality of life^7^. Furthermore, nocturia is correlated with an elevated probability of experiencing depressive symptoms^8,9^. Furthermore, there exists a significant correlation between cardiovascular disease and the prevalence of nocturia^10^. Research has demonstrated that the manifestation of nocturia serves as an initial indication of CKD and arises from a compromised capacity to concentrate urine^11^.

Based on a review of previous research, the association between the frequency of nocturnal urination and eGFR remains inconclusive. A study^12^ reported a significant association between CKD and the severity of nocturia. Furthermore, the findings of Minhang et al.^13^ indicated that higher eGFR was less likely to be linked with nocturia (odds ratio < 1). In a study conducted by Xuke et al.^14^, it was observed that patients with CKD exhibited significantly elevated nocturia score. Furthermore, the study identified lower eGFR and overweight as independent risk factors for nocturia. However, in a cross-sectional study^15^ involving 861 older adults residing in the community, no significant correlation was observed between nocturia and decreased eGFR when defining nocturia as two or more nocturnal urination. Considering the lack of clarity regarding the relationship between nocturnal urination frequency and eGFR, further investigation into their association is deemed imperative.

## Methods

### Study population

The present study utilized data from the NHANES spanning the years 2005 to 2018. This dataset provided valuable information pertaining to the frequency of nocturnal urination, the presence of prostate enlargement, and renal outcomes. Our investigation specifically focused on nocturnal urination frequency, eGFR, and urinary albumin to creatinine ratio (UACR). Supplementary Fig. 1 displays the flowchart illustrating the study design. Participants were included in the analysis only if they had complete data on all variables, including race/ethnicity, gender, age, fasting blood glucose, glycosylated hemoglobin (HbA1c), triglyceride (TG), total cholesterol (TCHOL), high-density lipoprotein (HDL), low density lipoprotein (LDL), uric acid, eGFR, creatinine (Cr), UACR, body mass index (BMI), previous medical history (hypertension, diabetes), history of smoking and alcohol consumption and psychological factor (item patient health questionnaire-9 (PHQ-9)). It is important to highlight that individuals with prostate enlargement were deliberately excluded from the study. The absence of ethical review was justified by the fact that all data used in the study were publicly accessible and fully anonymized. This report adheres to the reporting guidelines for cross-sectional studies outlined in the Strengthening the Reporting of Observational Studies in Epidemiology (STROBE)^16^.

### Measurement and definition of nocturia

Each participant’s nocturia data was obtained from the NHANES questionnaire on “During the past 30 days, how many times per night did you most typically get up to urinate, from the time you went to bed at night until the time you got up in the morning”. Following the guidelines set by the ICS, participants who reported urinating at least once during the night were diagnosed with nocturia.

### Measurement of eGFR

The eGFR measurement is not readily accessible within the NHANES database. Consequently, the Chronic Kidney Disease Epidemiology(CKD-EPI) equation, as developed by Levey^17^, was employed to estimate eGFR. Levey^17^ synthesized data from 10 studies to establish the CKD-EPI formula, which allows for the estimation of serum creatinine GFR. The formula underwent validation through data from 16 studies, demonstrating enhanced accuracy and reduced bias, particularly in the calculation of eGFR ≥ 60 mg/min.

### Covariates

NHANES collected data regarding the age, sex, and race (non-Hispanic white, non-Hispanic black, Mexican American, and other) of participants. During the physical examination phase of the survey, measurements of height, weight, and blood pressure were obtained. Medical professionals collected blood and urine samples, which were subsequently sent to the testing facility. The study also gathered the following covariates: fasting blood glucose, HbA1c, biochemical markers (ALT, AST, Cr and uric acid), lipids (TG, TCHOL, LDL and HDL), UACR, PHQ-9 score, past medical history (hypertension and diabetes) and history of smoking and alcohol consumption.

The BMI was determined by dividing the measured weight in kilograms by the measured height in meters squared^18^. This resulted in a categorical classification of BMI, with underweight defined as BMI < 18.5, normal weight as 18.5 ≤ BMI < 25, overweight as 25 ≤ BMI < 30, and obese as BMI ≥ 30.

Hypertension was determined based on the criteria of having a systolic blood pressure ≥ 140 mm Hg, or a diastolic blood pressure ≥ 90 mm Hg, measured on three consecutive occasions. Additionally, it encompassed individuals who were prescribed medication for hypertension or had received a professional diagnosis from a doctor or other healthcare provider. diabetes, on the other hand, was diagnosed when a physician informed the participant of their condition and prescribed hypoglycemic medication for the regulation of blood glucose levels.

Extract answers to the following questions: “In the past 12 months, on those days that you drank alcoholic beverages, on the average, how many drinks did you have?” and “Days have 5 or more drinks/past 12 month” and “Days per week, month, year?” and “Had at least 12 alcohol drinks/lifetime?”. Drinking severity was defined^19^ as (1) heavy alcohol user: ≥ 3 drinks per day for female, ≥ 4 drinks per day for male, binge drinking on 5 or more days per month. (2) moderate alcohol user: ≥ 2 drinks per day for female, ≥ 3 drinks per day for male, binge drinking ≥ 2 days per month. (3) mild alcohol user: ≥ 1 drinks per day for female, ≥ 2 drinks per day for male. (4) non-drinking was defined as answering no to the question “Had at least 12 alcohol drinks/lifetime?”.

Smoking is defined as answering “yes” to the following questions “Smoked at least 100 cigarettes in life?”. Or answer “every day” or “some days” to the question “Do you now smoke cigarettes?”. Otherwise, is defined as non-smoking.

In this study, the assessment of depressive symptoms was carried out utilizing the PHQ-9, a tool comprising nine items derived from the symptoms of depression outlined in the Diagnostic and Statistical Manual of Mental Disorders-IV (DSM-IV)^20^. For the purposes of this study, depressions deemed clinically significant were defined as those with PHQ-9 total score equal to or exceeding 10^21^.

### Statistical analysis

The principle of weighting served as the “lowest common denominator”. In this study, NHANES provided weights for fasting blood glucose samples, which were then combined with weights from all 8 cycles and followed NHANES guidelines for analysis. Linear regression analysis was employed to ascertain the association between frequency of nocturnal urination and eGFR. The ultimate model incorporates adjustments for significant confounding variables, which are determined by observing changes in effect estimates of more than 10% or regression coefficients of covariates with *p*-values below 0.1. A stratified analysis was conducted to examine the impact of nocturnal urination frequency on the continuous variable of eGFR. Additionally, stratified analyses and interaction tests were conducted for each covariate to assess their influence on the association between nocturnal urination frequency and eGFR. Finally, sensitivity analyses were conducted in diverse populations to ensure the robustness of the findings. The statistical software programs R (version 4.2.0) and Empower Stats (version 4.0) were employed to conduct all the analyses. Statistical significance was evaluated utilizing a two-sided significance level of 0.05.

## Results

Supplementary Table 1 presents demographic information and descriptive statistics related to nocturnal urination frequency for the 12,265 NHANES individuals who participated in this study. In addition, Table 1 presents weighted demographic information and descriptive statistics related to frequency of nocturnal urination. Among these individuals, the highest number of individuals reported urinating once at night. Briefly, the number of individuals with no nocturnal urination, as well as those with 1, 2, 3, 4, and 5 or more episodes of nocturnal urination was 3720, 4705, 2301, 979, 327 and 233, respectively. Furthermore, the majority of the study population included individuals with CKD stages 1 and 2. In comparison to individuals who experienced voided 0-time or 1-time during night, experienced voided 2 or more times exhibited elevated levels of age, fasting blood glucose, HbA1C, ALT, TG, HDL, BMI, and UACR. Conversely, individuals with a higher frequency of nighttime urination demonstrated a lower eGFR. Additionally, an escalation in the frequency of nocturia was associated with an increased prevalence of smoking, drinking, hypertension, diabetes, CKD, and depression. In populations exhibiting varying prognostic risks for CKD, a notable rise in the percentage of individuals experiencing voiding more than three times at night was observed with in the very high risk, high risk and moderate risk groups. Nevertheless, no statistically significant differences were found in the levels of AST, uric acid, TCHOL, and LDL across different frequency groups of nocturnal urination (*P* > 0.05).

**Table 1:** Weighted baseline characteristics of study participants according to frequency of nocturnal urination.

	Nocturnal urination frequency						
	0	1	2	3	4	≥ 5	*P* value
N	3558	4507	2183	925	312	218	
Age (years)	38.0 (28.0, 51.0)	48.0 (34.0, 61.0)	54.0 (41.0, 66.0)	58.0 (46.0, 68.0)	59.0 (46.0, 71.0)	51.0 (39.0, 63.0)	< 0.0001
Sex [%(N)]							< 0.0001
Female	46.3 (1668)	52.0 (2323)	57.1 (1229)	56.3 (516)	64.3 (190)	51.9 (119)	
Male	53.7 (1890)	48.0 (2184)	42.9 (954)	43.7 (409)	35.7 (122)	48.1 (99)	
Race [%(N)]							< 0.0001
Non-Hispanic white	67.6 (1524)	70.3 (2049)	64.8 (866)	61.4 (348)	52.1 (98)	52.5 (63)	
Non-Hispanic black	8.0 (532)	10.0 (822)	14.6 (531)	17.9 (247)	23.8 (98)	23.5 (71)	
Mexican American	9.3 (594)	7.5 (631)	9.0 (352)	8.4 (147)	8.6 (48)	10.6 (38)	
Other	15.1 (908)	12.3 (1005)	11.5 (434)	12.3 (183)	15.5 (68)	13.4 (46)	
eGFR (mL/min/1.73 m^2^)	101.6 (87.8, 115.0)	96.1 (81.3, 110.3)	92.2 (76.2, 107.3)	90.3 (73.4, 104.8)	86.2 (73.4, 101.8)	89.5 (78.9, 106.6)	< 0.0001
Cr (mmol/L)	75.1 (63.6, 86.6)	74.3 (63.6, 85.8)	72.5 (62.8, 87.5)	73.4 (62.8, 87.5)	72.5 (61.9, 84.0)	76.0 (63.6, 91.9)	0.2928
UACR (mg/g)	5.9 (4.1, 9.5)	6.3 (4.2, 11.3)	7.4 (4.8, 14.1)	8.1 (5.0, 15.7)	10.8 (5.4, 26.1)	8.7 (4.9, 24.0)	< 0.0001
Fasting blood glucose (mmol/L)	5.4 (5.1, 5.8)	5.6 (5.2, 6.0)	5.7 (5.2, 6.3)	5.8 (5.3, 6.4)	5.8 (5.3, 6.5)	5.8 (5.2, 6.5)	< 0.0001
HbA1C (%)	5.3 (5.1, 5.6)	5.5 (5.2, 5.8)	5.5 (5.3, 6.0)	5.6 (5.4, 6.0)	5.8 (5.4, 6.1)	5.6 (5.3, 6.1)	< 0.0001
ALT (U/L)	21.0 (16.0, 29.0)	21.0 (16.0, 28.0)	21.0 (16.0, 28.0)	19.0 (15.0, 27.0)	19.0 (15.0, 25.0)	24.0 (16.0, 31.0)	0.0266
AST (U/L)	22.0 (19.0, 27.0)	22.0 (19.0, 27.0)	22.0 (19.0, 27.0)	22.0 (19.0, 27.0)	22.0 (18.0, 25.0)	23.0 (19.0, 29.0)	0.1813
Uric acid (umol/L)	321.2 (267.7, 374.7)	321.2 (267.7, 374.7)	321.2 (261.7, 380.7)	327.1 (267.7, 380.7)	315.2 (255.8, 374.7)	321.2 (273.6, 374.7)	0.7409
TG (umol/L)	1.1 (0.7, 1.6)	1.1 (0.8, 1.6)	1.1 (0.8, 1.7)	1.2 (0.8, 1.7)	1.2 (0.9, 1.8)	1.3 (0.8, 2.0)	0.0025
TCHOL (umol/L)	4.8 (4.2, 5.6)	4.9 (4.2, 5.6)	4.8 (4.2, 5.5)	4.9 (4.2, 5.6)	5.0 (4.3, 5.7)	4.9 (4.4, 5.6)	0.5655
HDL (umol/L)	1.3 (1.1, 1.6)	1.3 (1.1, 1.7)	1.3 (1.1, 1.7)	1.3 (1.1, 1.6)	1.3 (1.1, 1.6)	1.2 (1.0, 1.5)	0.0045
LDL (umol/L)	2.9 (2.4, 3.5)	2.9 (2.3, 3.5)	2.8 (2.3, 3.5)	2.9 (2.2, 3.5)	3.1 (2.4, 3.7)	2.9 (2.3, 3.5)	0.1677
BMI (kg/m^2^)	27.0 (23.5, 31.3)	27.8 (24.2, 32.4)	29.0 (25.0, 34.4)	29.4 (25.5, 34.6)	29.8 (25.4, 35.1)	29.4 (25.8, 34.6)	< 0.0001
Drinking [%(N)]							< 0.0001
Never	9.4 (458)	9.7 (558)	13.4 (376)	14.0 (150)	22.4 (70)	16.2 (38)	
Mild	47.2 (1660)	51.8 (2300)	51.1 (1124)	56.1 (505)	46.9 (159)	49.1 (110)	
Moderate	18.7 (609)	18.2 (734)	16.6 (308)	13.0 (117)	12.5 (35)	12.4 (22)	
Heavy	24.7 (831)	20.3 (915)	18.9 (384)	16.8 (153)	18.2 (48)	22.3 (48)	
Smoking [%(N)]							< 0.0001
No	59.1 (2125)	56.4 (2549)	52.9 (1196)	46.0 (459)	48.5 (154)	52.0 (100)	
Yes	40.9 (1433)	43.6 (1958)	47.1 (987)	54.0 (466)	51.5 (158)	48.0 (118)	
Hypertension [%(N)]							< 0.0001
No	74.4 (2578)	62.7 (2666)	49.2 (1013)	43.0 (332)	40.5 (112)	42.6 (71)	
Yes	25.6 (980)	37.3 (1841)	50.8 (1171)	57.0 (593)	59.5 (200)	57.4 (147)	
Diabetes [%(N)]							< 0.0001
No	94.8 (3299)	91.0 (3974)	84.5 (1781)	78.1 (677)	78.0 (226)	76.0 (143)	
Yes	5.2 (239)	9.0 (533)	15.5 (402)	21.9 (248)	22.0 (86)	24.0 (75)	
CKD [%(N)]							< 0.0001
No	92.1 (3203)	87.6 (3837)	80.5 (1698)	78.5 (658)	72.0 (219)	73.7 (136)	
Yes	7.9 (355)	12.4 (670)	19.5 (485)	21.5 (267)	28.0 (93)	26.3 (82)	
CKD prognosis risk [%(N)]							< 0.0001
Very high risk	0.5 (28)	1.0 (70)	1.8 (59)	3.2 (42)	4.2 (16)	4.6 (15)	< 0.0001
High risk	0.9 (53)	1.9 (113)	3.8 (107)	5.8 (65)	4.9 (15)	4.6 (16)	
Moderately increased risk	6.4 (274)	9.4 (487)	13.9 (319)	12.6 (160)	18.9 (62)	17.2 (51)	
Low risk	92.1 (3203)	87.6 (3837)	80.5 (1698)	78.5 (658)	72.0 (219)	73.7 (136)	
CKD stages [%(N)]							< 0.0001
Stage 1	71.8 (2586)	60.4 (2797)	53.5 (1170)	51.2 (443)	46.0 (140)	48.1 (105)	
Stage 2	25.0 (824)	34.2 (1415)	37.4 (781)	38.4 (360)	43.1 (129)	43.0 (85)	
Stage 3	2.9 (135)	5.0 (264)	8.2 (202)	9.4 (110)	9.6 (39)	7.3 (23)	
Stage 4	0.2 (7)	0.3 (22)	0.7 (26)	0.7 (9)	1.2 (3)	0.3 (1)	
Stage 5	0.1(6)	0.1 (9)	0.1 (4)	0.3 (3)	0.2 (1)	1.4 (4)	
PHQ-9 score [%(N)]							< 0.0001
< 10	95.4 (3365)	93.9 (4198)	89.5 (1951)	81.5 (760)	82.8 (254)	75.6 (159)	
≥ 10	4.6 (193)	6.1 (309)	10.5 (232)	18.5 (165)	17.2 (58)	24.4 (59)	

HbA1c, glycosylated hemoglobin; ALT, glutamic pyruvic transaminase; AST, glutamic oxaloacetic transaminase; TG, triglyceride; TCHOL, total cholesterol; HDL, high-density lipoprotein; LDL, low density lipoprotein; BMI, body mass index; eGFR, estimated glomerular filtration rate; UACR, Urinary albumin to creatinine ratio; CKD, chronic kidney disease; PHQ-9, item patient health questionnaire-9.

Continuous variables were represented using survey-weighted medians (Q1, Q3), and *p*-values were derived through survey-weighted linear regression (svyglm). Categorical variables were presented as survey-weighted percentages (95% CI), and *p*-values were computed using survey-weighted Chi-square tests (svytable).

### Nocturia is associated with a decrease in eGFR

The initial examination focused on the association between the gathered covariates and eGFR, as presented in supplement Table 2. Univariate analysis demonstrated a significant association between age, elevated fasting blood glucose, HbA1c, uric acid, lipids, BMI, and increased frequency of nocturnal urination with a decline in eGFR. Furthermore, smoking, hypertension, and diabetes were also found to be associated with a decrease in eGFR. These findings align with previous research studies^22–25^. This study examines the potential use of the PHQ-9 score as a diagnostic tool for depression, considering the established relationship between depressive symptoms and the risk of rapid decline in eGFR, end-stage renal disease and acute kidney injury^26^. Furthermore, it acknowledges the documented association between nocturia and increased likelihood of depression^8,9,27^. However, it is important to note that this study did not observe a significant association between depression and eGFR.

To evaluate the independent impact of nocturia on eGFR, we defined nocturia as the occurrence of nocturnal urination at least once. As indicated in Table 2, nocturia was found to be significantly associated with a reduction of 7.3 ml/min/1.73 m^2^ in eGFR when compared to individuals without nocturia, without any adjustments for confounding variables. In model II, after adjusting for important covariates such as CKD, CKD prognosis risk, fasting blood glucose, HbA1c, ALT, AST, Cr, uric acid, TG, HDL, drinking habits, smoking status, hypertension, BMI, and PHQ-9 score, linear regression analysis revealed that nocturia was still associated with a decrease of 4.0 ml/min/1.73 m^2^ in eGFR compared to individuals without nocturia.

**Table 2:** Relationship between nocturia/nocturnal urination frequency and eGFR in study population.

Outcome	Crude model β (95% CI) *P* value	Model I β (95% CI) *P* value	Model II β (95% CI) *P* value
Nocturia vs. non-nocturia(ref.)	− 7.3 (− 8.1, − 6.5) < 0.001	1.1 (0.5, 1.7) < 0.001	− 4.0 (− 4.6, − 3.4) < 0.001
Nocturnal urination frequency (continuous)	− 3.5 (− 3.9, − 3.1) < 0.001	0.3 (0.0, 0.5) 0.048	− 2.0 (− 2.2, − 1.7) < 0.001
Nocturnal urination frequency (categorical)			
0	Ref	Ref	Ref
1	− 5.5 (− 6.4, − 4.6) < 0.001	1.0 (0.4, 1.6) 0.002	− 3.1 (− 3.7, − 2.5) < 0.001
2	− 9.3 (− 10.5, − 8.2) < 0.001	1.4 (0.5, 2.2) 0.001	− 5.4 (− 6.2, − 4.6) < 0.001
3	− 11.4 (− 13.1, − 9.7) < 0.001	1.7 (0.4, 2.9) 0.007	− 6.4 (− 7.6, − 5.3) < 0.001
4	− 13.0 (− 16.0, − 10.0) < 0.001	0.6 (− 1.5, 2.8) 0.559	− 8.6 (− 10.6, − 6.5) < 0.001
≥ 5	− 9.9 (− 13.3, − 6.4) < 0.001	− 2.0 (− 4.4, 0.4) 0.108	− 4.0 (− 6.3, − 1.7) < 0.001

Crude model adjust for: None. Model I adjust for: sex; race; age. Model II adjust for: Model I + CKD; CKD prognosis risk; HbA1c; ALT; AST; Cr; TG; HDL; uric acid; drinking; smoking; hypertension; BMI; PHQ-9.

Furthermore, in order to provide a more comprehensive understanding of the independent impact of nocturnal urination frequency on eGFR, we employed measures to control for potential confounding variables and conducted a linear regression analysis as presented in Table 2. Our findings indicate that a single increase in the frequency of nocturnal urination is associated with a decrease in eGFR by 3.5 mL/min/1.73 m^2^ (95% CI − 3.9, − 3.1) when not accounting for other confounders. However, after adjusting for these confounding factors, we observed that a one-time increase in nocturnal urination frequency is linked to a reduction in eGFR by 2.0 mL/min/1.73 m^2^ (95% CI − 2.2, − 1.7).

Subsequently, in order to provide a more comprehensive understanding of the association between nocturnal urinary frequency and eGFR, we conducted an analysis of nocturnal urinary frequency using categorical variables. In the absence of any adjustments for variables, our findings indicate that the population with a frequency of one nocturnal urination experienced a decrease in eGFR of 5.5 mL/min/1.73 m^2^ (95% CI − 6.4, − 4.6) in comparison to those with no nocturnal urination. Similarly, individuals who voided 2, 3, 4, and ≥ 5 times during the night exhibited a decrease in eGFR of 9.3 mL/min/1.73 m^2^ (95% CI − 10.5, − 8.2), 11.4 mL/min/1.73 m^2^ (95% CI − 13.1, − 9.7), 13.0 mL/min/1.73 m^2^ (95% CI − 16.0, − 10.0), and 9.9 mL/min/1.73 m^2^ (95% CI − 13.3, − 6.4), respectively. Furthermore, following adjustment for various confounding variables, our analysis revealed a significant decline in eGFR of 3.1 mL/min/1.73 m^2^ among individuals who voided once during the nocturnal period, as compared to those who did not void at all. Moreover, we observed a progressive decrease in eGFR of 5.4 mL/min/1.73 m^2^ (95% CI − 6.2, − 4.6), 6.4 mL/min/1.73 m^2^ (95% CI − 7.6, − 5.3), 8.6 mL/min/1.73 m^2^ (95% CI − 10.6, − 6.5), and 4.0 mL/min/1.73 m^2^ (95% CI − 6.3, − 1.7) for individuals who voided 2, 3, 4 and ≥ 5 times during the nocturnal period, respectively.

### Investigation of the association between nocturia and eGFR in different populations

**Figure 1:**
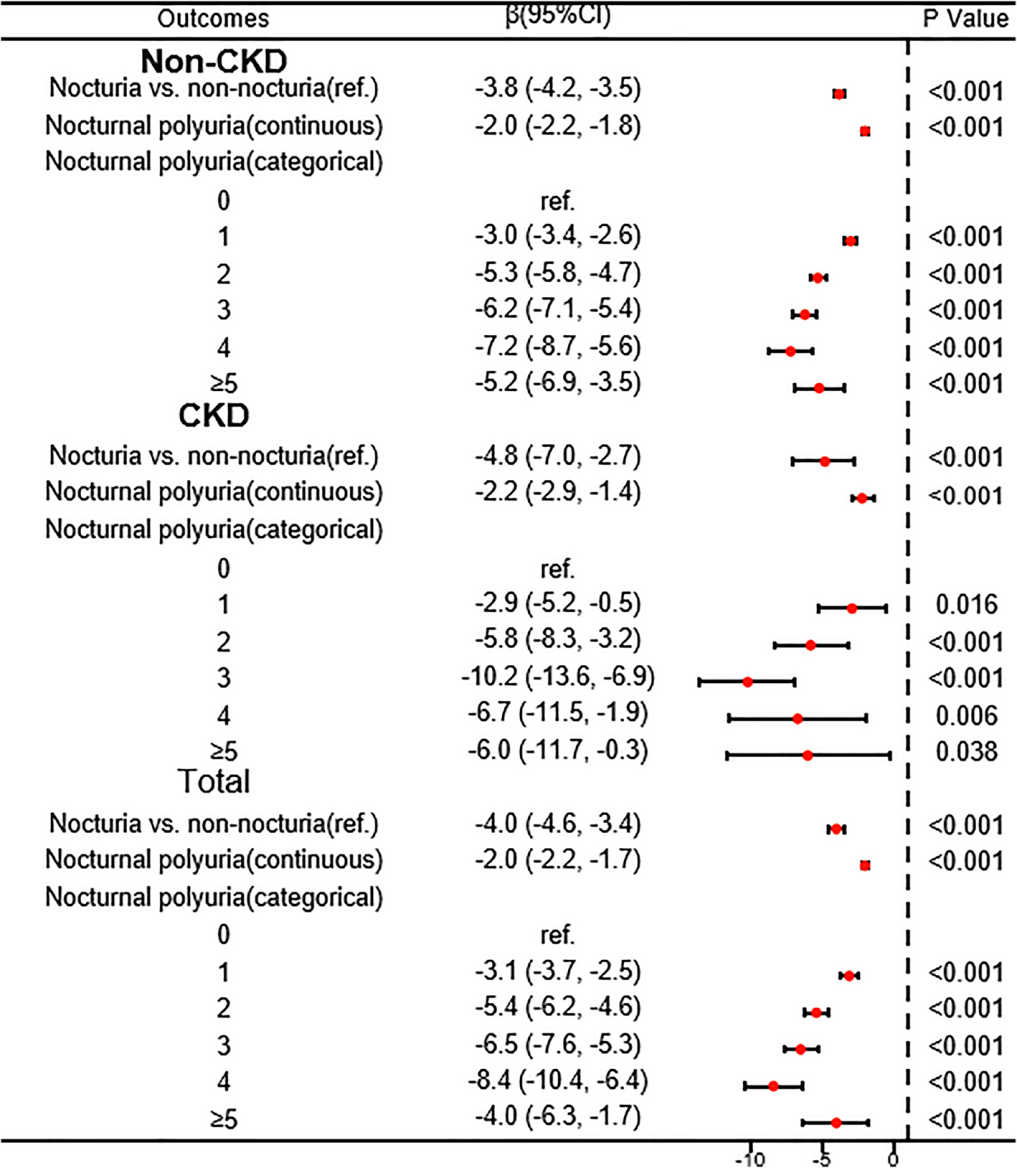
Forest plot showing the association of nocturia/nocturnal urination frequency with eGFR in CKD and non-CKD populations. The results of the analysis were adjusted for sex; race; age; HbA1c; ALT; AST; Cr; uric acid; TG; HDL; drinking; smoking; hypertension; BMI; PHQ-9.

**Figure 2:**
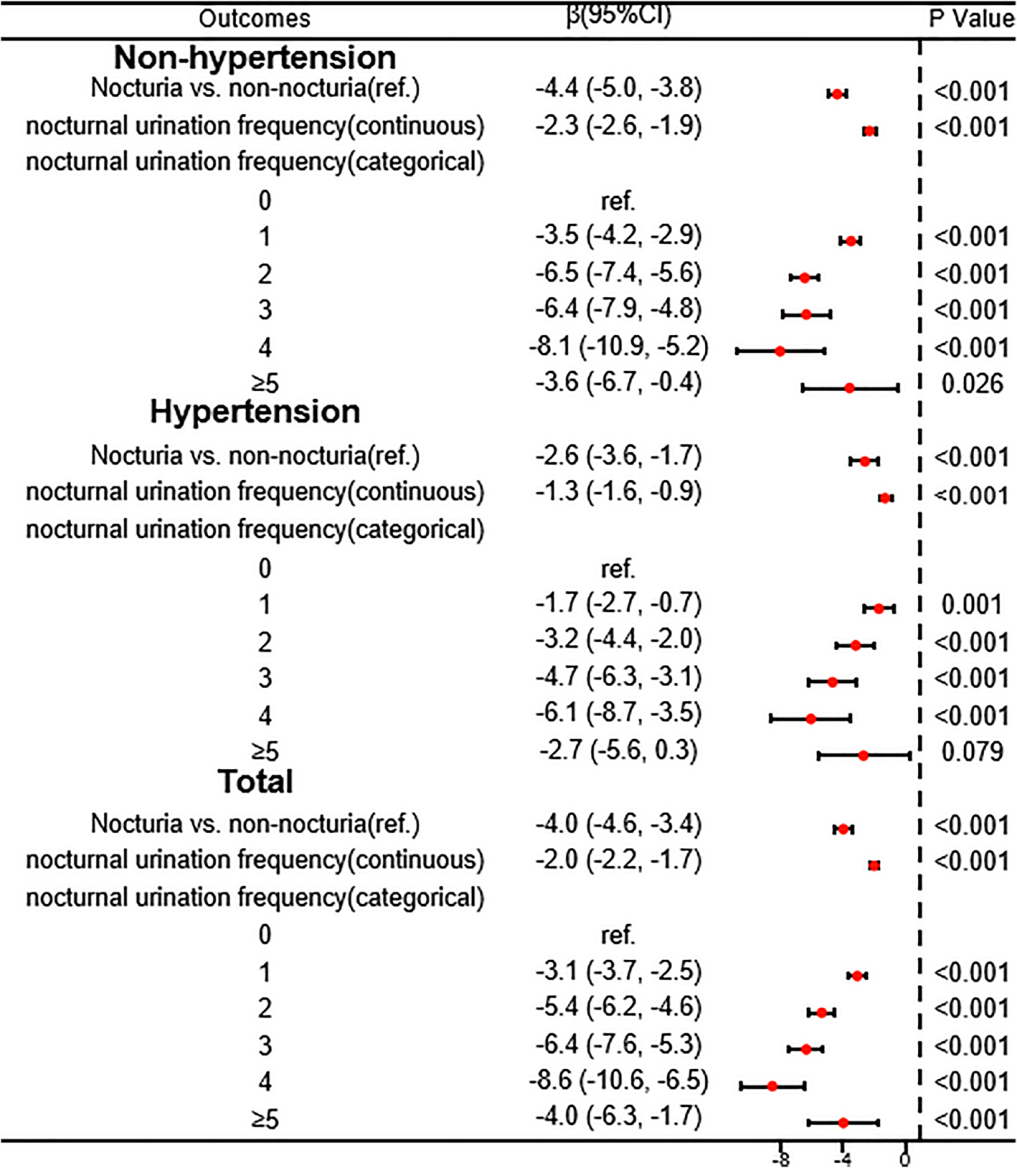
Forest plot showing the association of nocturia/nocturnal urination frequency with eGFR in varying hypertensive state. The results of the analysis were adjusted for sex; race; age; HbA1c; ALT; AST; Cr; uric acid; TG; HDL; drinking; smoking; BMI; PHQ-9; CKD; CKD prognosis risk.

**Figure 3:**
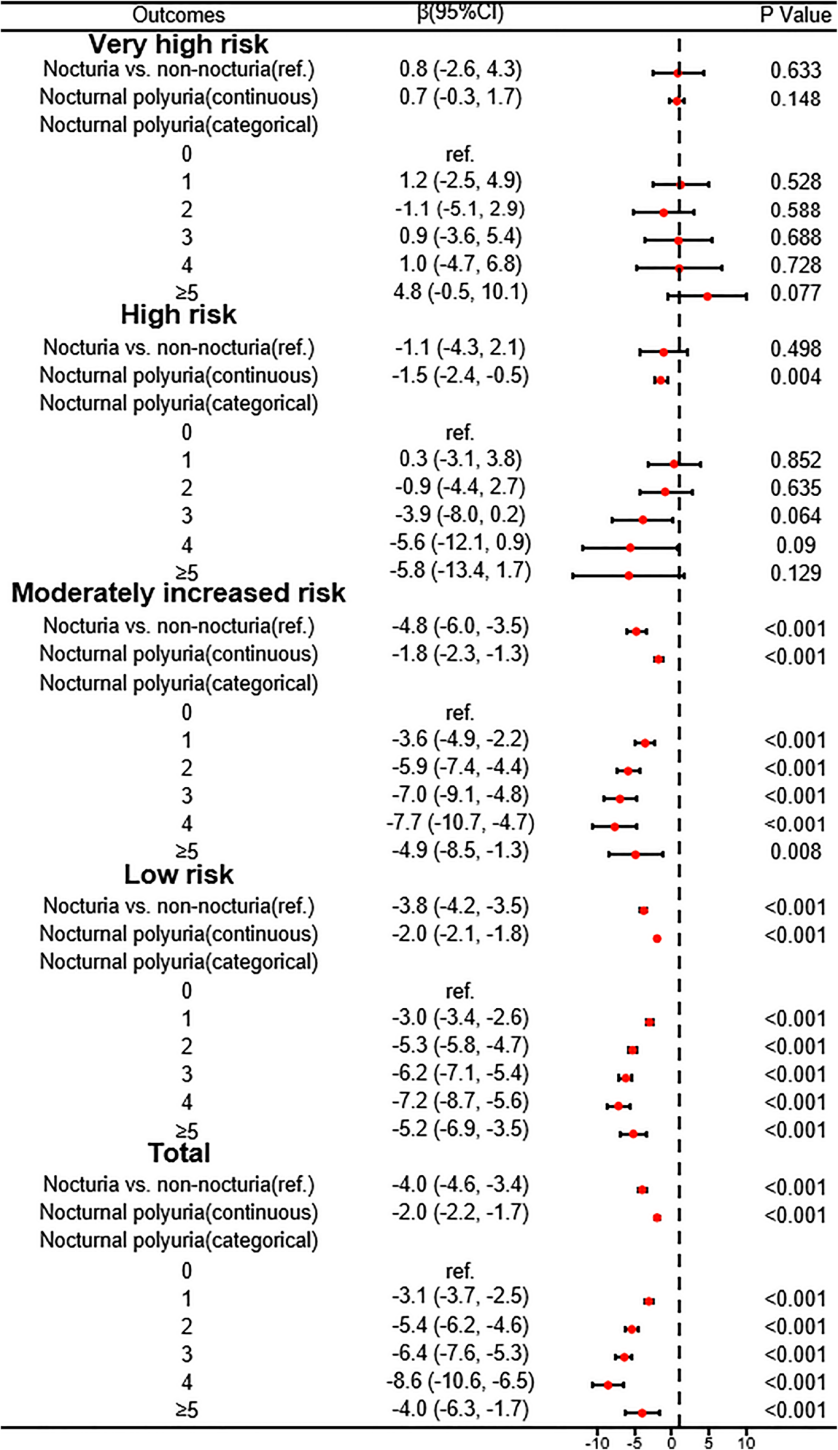
Forest plot showing the association of nocturia/nocturnal urination frequency with eGFR in varying degrees of CKD prognostic risk. The results of the analysis were adjusted for sex; race; age; HbA1c; ALT; AST; Cr; uric acid; TG; HDL; drinking; smoking; hypertension; BMI; PHQ-9.

According to the criteria established by Kidney Disease Improving Global Outcomes (KDIGO)^28^, the population was divided into two groups: those with CKD and those without CKD. This division was based on an eGFR of less than 60 mL/min/1.73 m^2^ and a UACR of 30 mg/g or higher. The eGFR and UACR were used to assess the prognostic risk of CKD in the participants. In order to determine the factors that influenced the impact of nocturnal urination frequency on eGFR, we examined the interaction of various confounding variables. Our analysis revealed a significant interaction between the frequency of nocturnal urination and hypertensive status, CKD status, and CKD prognostic risk (interaction *P* = 0.0497, 0.029, 0.0005, respectively) (see supplement Table 3). Consequently, a stratified analysis was conducted on various populations affected by CKD, populations with hypertension, and populations at risk for CKD prognosis. Figure 1 demonstrates a consistent association between eGFR and nocturia as well as nocturnal urination frequency in both CKD and non-CKD populations. Likewise, this association remained consistent in populations with and without hypertension, as depicted in Fig. 2. Furthermore, Fig. 3 reveals disparate findings among different prognostic risk populations for CKD. Our study revealed a significant correlation between the frequency of nocturia/nocturnal urination and a decline in eGFR among individuals classified as intermediate risk and low risk. However, no such association was observed in the very high risk and high risk groups.

## Discussion

In a comprehensive analysis of adult US citizens utilizing the NHANES database, an association was observed between nocturia/heightened frequency of nocturnal urination and diminished eGFR. This study stands as the most extensive investigation to date, demonstrating the presence of such a correlation within the American population. The outcomes align with a pathophysiological mechanism of nocturia, thereby indicating that it may serve as an indicator of renal disease^29–31^. Several early studies^32,33^ have investigated the etiology of nocturia, yet there has been a limited emphasis on exploring the correlation between nocturia and renal function. Historically, nocturia has been regarded as an inconsequential phenomenon, resulting in a scarcity of patients seeking medical intervention due to perceiving it as a natural consequence of aging^34^. Our research findings serve as a compelling alert to both physicians and patients, highlighting the association between nocturia and reduced eGFR. Consequently, this serves as a reminder to healthcare professionals to exercise vigilance regarding alterations in renal function when confronted with patients presenting with nocturia.

Our study revealed that, after accounting for confounding variables, the outcomes of the linear regression analysis demonstrated that a single increase in nocturnal voiding frequency resulted in a decrease of 2.0 mL/min/1.73 m^2^ in eGFR. In comparison to individuals with a nocturnal urinary frequency of 0, those who voided 1, 2, 3, 4, and ≥ 5 times during the night experienced a decrease in eGFR of 3.1, 5.4, 6.4, 8.6 and 4.0 mL/min/1.73 m^2^, respectively. Nocturia was found to be associated with a decrease in eGFR of 4.0 mL/min/1.73 m^2^ when compared to individuals without nocturia. These findings suggest a correlation between nocturia, increased frequency of nocturnal urination, and lower eGFR. There is compelling evidence indicating a strong correlation between nocturia and renal function. Empirical findings suggest that nocturia emerges as an early indicator of CKD when renal function declines, leading to a reduced ability to concentrate urine. Certain researchers propose that osmotic diuresis, rather than free-water diuresis, is responsible for plays a role in nocturia and impaired renal function in CKD^11,32^. In addition, in individuals with CKD, nocturia, may serve as a potential marker for diminished renal tubular function^35^. While prior research has demonstrated a connection between nocturia and renal function, our study represents the inaugural investigation into the correlation between nocturia and eGFR. Consequently, this finding serves as a valuable prompt for clinicians to be attentive to patients who report experiencing one or more episodes of nocturnal urination per night.

Numerous diseases have been found to be linked with the occurrence of nocturia. Research has demonstrated a significant 39% rise in the prevalence of nocturia among individuals classified as obese, with a BMI over 30 kg/m^2^, in comparison to those who are non-obese^36^. Subsequently, individuals diagnosed with diabetes were found to have a 49% higher likelihood of developing nocturia^37^. Moreover, advancing age has been strongly associated with the occurrence of nocturia^38^. Specifically, nocturia is reported by only 0.4% of adults below the age of 40, whereas the prevalence increases to 11.5% among individuals aged 60 and above^38^. Additionally, untreated hypertensive patients were found to be 39% more likely to report nocturia compared to normotensive men^39^. Hence, the risk factors associated with nocturia encompass the process of aging, hypertension, diabetes, and obesity^36,37,40,41^, all of which contribute to the development of CKD^42–45^. In order to evaluate the influence of these factors on the relationship between nocturia and eGFR, an interaction test was conducted. The findings revealed that hypertension significantly impacted the association between nocturia and eGFR, potentially due to the occurrence of hypertensive diuresis^46^. In the present study, a sensitivity analysis was conducted, revealing that the association between nocturia and eGFR was consistent across both non-hypertensive and hypertensive populations.

In conclusion, our findings indicate a significant correlation between that nocturia and heightened frequency of nocturnal urination with decreased eGFR, a relationship that holds true for both CKD and non-CKD, hypertensive and non-hypertensive cohorts. Nevertheless, this association does not persist uniformly across various CKD prognostic risk populations, specifically the very high risk and high risk groups, for which our conclusions are not applicable. This finding indicates that the association between nocturia and heightened frequency of nocturnal urination, as well as eGFR, becomes insignificant once renal function reaches a certain level of impairment. This observation serves as a reminder for healthcare professionals that nocturia should be given more attention when it manifests in individuals with mild renal function abnormalities.

Our study possesses several notable strengths. Firstly, the utilization of a population-based strategy, multi-stage probability sampling, and a substantial sample size greatly enhance the generalizability of our findings. Secondly, we have conducted the first investigation into the correlation between nocturia and eGFR, thereby raising awareness among physicians regarding renal function in patients experiencing nocturia. Lastly, we have systematically stratified additional factors based on their influence on nocturia and evaluated their interactions with nocturia, thereby providing a potential mechanistic understanding of the impact of nocturia on renal function.

It is crucial to recognize the limitations of our study. Due to its cross-sectional nature, our investigation only allows for the identification of an association between nocturia exposure and eGFR, rather than establishing causal evidence. Furthermore, the diagnosis of nocturia relied on participants completing a voiding diary. However, the data regarding nocturnal urinary frequency were obtained from self-reported individuals who did not comply with a voiding diary, thereby necessitating the need to validate of our conclusions in individuals who experience increased nocturnal urination and diligently maintain a bladder diary. Furthermore, our findings were not evident in cases where the prognostic risk of CKD was deemed high or very high, which could potentially be attributed to the limited number of individuals falling within these specific risk categories. In order to achieve more precise findings, our objective is to conduct future research endeavors that encompass larger sample sizes within these two cohorts. Additionally, the data collection process was afflicted by uncertainty, leading to a significant amount of missing data in this study. To ensure the reliability of the results, the population with incomplete data was intentionally excluded from this analysis. Furthermore, the potential influence of prostate enlargement and overactive bladder syndrome on the outcomes of our study warrants consideration. In order to address this, participants who self-reported prostate enlargement were deliberately excluded from our study. However, the lack of available data pertaining to individuals with overactive bladder syndrome hindered our ability to definitively ascertain its impact on the results. Consequently, it is important to acknowledge that our study may not be entirely representative of the entire population of the US. Lastly, the limited availability of comprehensive drug-related data in the NHANES database posed a hindrance to our capacity to determine the impact of medications, including diuretics, on our findings. It is expected that forthcoming research endeavors will incorporate supplementary covariates to augment the credibility of our conclusion.

## Conclusion

The presence of nocturia and heightened frequency of nocturnal urination exhibited a correlation with a decline in eGFR. Nevertheless, this association was not observed within the subsets of CKD patients classified as having a very high prognostic risk or high risk.

### Supplementary Information

Supplementary Figure 1.

Supplementary Tables.

## Data Availability

The data that support the findings of this study are openly available in National Health and Nutrition Examination Survey at https://www.cdc.gov/nchs/nhanes/index.htm. or it can be requested from the corresponding author and is available upon reasonable request.
