# Supplementary figures and images for: Association of nocturia of self-report with estimated glomerular filtration rate: a cross-sectional study from the NHANES 2005–2018
Source: Sci Rep. 2023 Aug 25;13:13924. doi: 10.1038/s41598-023-39448-0 (PMC10457317; doi:10.1038/s41598-023-39448-0)

**Supplement figure 1 Flowchart for inclusion of study participants.**


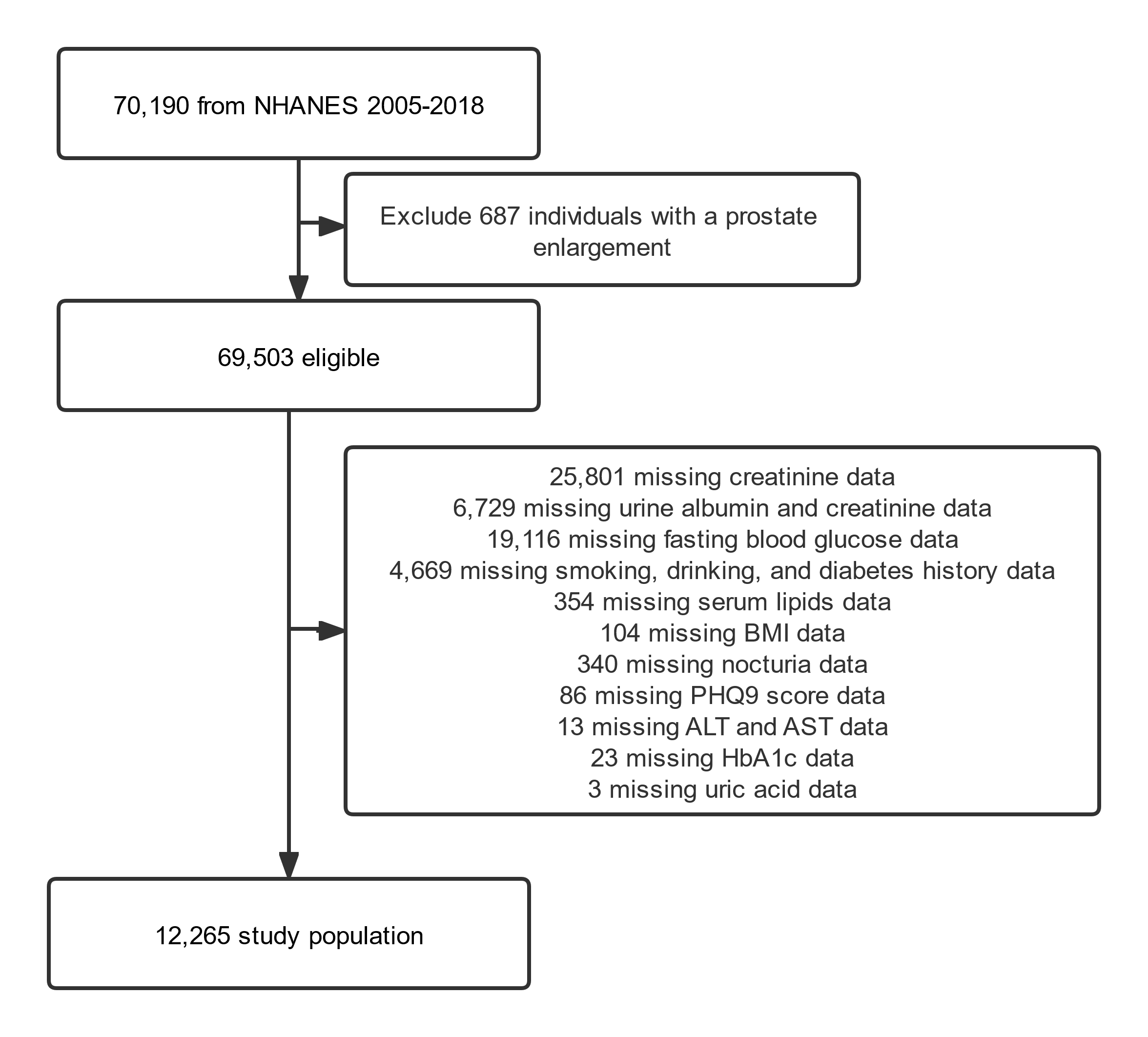

Supplement: Supplementary file 1 — Supplementary Figure 1. [file 41598_2023_39448_MOESM1_ESM.docx]
