# Supplementary material for: Association of nocturia of self-report with estimated glomerular filtration rate: a cross-sectional study from the NHANES 2005–2018
Source: Sci Rep. 2023 Aug 25;13:13924. doi: 10.1038/s41598-023-39448-0 (PMC10457317; doi:10.1038/s41598-023-39448-0)
Supplement: Supplementary file 2 — Supplementary Tables. [file 41598_2023_39448_MOESM2_ESM.docx]

**Supplement Table 1 Baseline characteristics of study participants according to frequency of nocturnal urination**

|  | nocturnal urination frequency | | | | | | |
| --- | --- | --- | --- | --- | --- | --- | --- |
|  | 0 | 1 | 2 | 3 | 4 | ≥5 | P Value |
| N | 3720 | 4705 | 2301 | 979 | 327 | 233 |  |
| Age (years) | 40.0 (29.0, 54.0) | 50.0 (36.0, 63.0) | 57.0 (43.0, 69.0) | 61.0 (49.0, 71.0) | 62.0 (49.0, 72.0) | 59.0 (44.0, 69.0) | <0.001 |
| Sex [N (%)] |  |  |  |  |  |  | <0.001 |
| Female | 1729 (46.5%) | 2413 (51.3%) | 1288 (56.0%) | 552 (56.4%) | 200 (61.2%) | 126 (54.1%) |  |
| Male | 1991 (53.5%) | 2292 (48.7%) | 1013 (44.0%) | 427 (43.6%) | 127 (38.8%) | 107 (45.9%) |  |
| Race [N (%)] |  |  |  |  |  |  | <0.001 |
| Non-Hispanic white | 1597 (42.9%) | 2151 (45.7%) | 905 (39.3%) | 372 (38.0%) | 101 (30.9%) | 72 (30.9%) |  |
| Non-Hispanic black | 562 (15.1%) | 875 (18.6%) | 569 (24.7%) | 258 (26.4%) | 106 (32.4%) | 73 (31.3%) |  |
| Mexican American | 616 (16.6%) | 646 (13.7%) | 375 (16.3%) | 153 (15.6%) | 48 (14.7%) | 40 (17.2%) |  |
| Other | 945 (25.4%) | 1033 (22.0%) | 452 (19.6%) | 196 (20.0%) | 72 (22.0%) | 48 (20.6%) |  |
| eGFR (mL/min/1.73 m^2^) | 102.6 (88.3, 116.1) | 96.9 (81.3, 111.1) | 91.9 (75.3, 107.2) | 88.5 (70.1, 104.7) | 86.2 (70.1, 101.2) | 90.0 (73.7, 104.3) | <0.001 |
| Cr (mmol/L) | 74.3 (61.9, 86.6) | 73.4 (62.8, 86.6) | 73.4 (62.8, 89.3) | 75.1 (62.8, 90.2) | 72.5 (62.3, 89.3) | 75.1 (61.9, 92.8) | <0.001 |
| UACR (mg/g) | 6.2 (4.2, 10.4) | 6.8 (4.4, 12.8) | 8.3 (5.1, 17.8) | 9.3 (5.5, 23.0) | 10.8 (5.6, 26.8) | 11.4 (5.6, 45.2) | <0.001 |
| Fasting blood glucose (mmol/L) | 5.5 (5.1, 5.9) | 5.6 (5.2, 6.1) | 5.7 (5.3, 6.4) | 5.9 (5.4, 6.8) | 5.9 (5.4, 6.7) | 5.9 (5.3, 6.7) | <0.001 |
| HbA1C (%) | 5.4 (5.2, 5.7) | 5.5 (5.3, 5.9) | 5.6 (5.3, 6.1) | 5.8 (5.4, 6.3) | 5.8 (5.4, 6.3) | 5.8 (5.4, 6.4) | <0.001 |
| ALT (U/L) | 21.0 (16.0, 29.0) | 21.0 (16.0, 28.0) | 20.0 (16.0, 27.0) | 20.0 (15.0, 26.0) | 19.0 (15.0, 24.0) | 21.0 (16.0, 29.0) | 0.028 |
| AST (U/L) | 22.0 (19.0, 27.0) | 23.0 (19.0, 27.0) | 22.0 (19.0, 27.0) | 23.0 (19.0, 28.0) | 21.0 (18.0, 25.0) | 22.0 (19.0, 29.0) | 0.030 |
| Uric acid (umol/L) | 321.2 (267.7, 374.7) | 321.2 (267.7, 380.7) | 321.2 (267.7, 386.6) | 327.1 (273.6, 386.6) | 315.2 (279.6, 386.6) | 327.1 (273.6, 386.6) | 0.002 |
| TG (umol/L) | 1.1 (0.8, 1.6) | 1.1 (0.8, 1.6) | 1.2 (0.8, 1.7) | 1.2 (0.9, 1.7) | 1.1 (0.9, 1.6) | 1.2 (0.8, 1.9) | <0.001 |
| TCHOL (umol/L) | 4.8 (4.2, 5.6) | 4.9 (4.2, 5.6) | 4.9 (4.2, 5.5) | 4.8 (4.2, 5.5) | 5.0 (4.3, 5.7) | 4.8 (4.1, 5.6) | 0.133 |
| HDL (umol/L) | 1.3 (1.1, 1.6) | 1.3 (1.1, 1.6) | 1.3 (1.1, 1.7) | 1.3 (1.1, 1.6) | 1.4 (1.1, 1.7) | 1.3 (1.1, 1.6) | 0.002 |
| LDL (umol/L) | 2.9 (2.3, 3.5) | 2.9 (2.3, 3.5) | 2.8 (2.3, 3.4) | 2.8 (2.2, 3.5) | 3.0 (2.3, 3.6) | 2.8 (2.2, 3.5) | 0.009 |
| BMI (kg/m^2^) | 27.1 (23.5, 31.3) | 27.8 (24.3, 32.2) | 28.8 (25.1, 34.1) | 29.7 (25.5, 34.4) | 30.2 (26.0, 35.5) | 29.7 (25.6, 34.7) | <0.001 |
| Drinking [N (%)] |  |  |  |  |  |  | <0.001 |
| Never | 479 (12.9%) | 578 (12.3%) | 388 (16.9%) | 161 (16.4%) | 75 (22.9%) | 41 (17.6%) |  |
| Mild | 1740 (46.8%) | 2394 (50.9%) | 1179 (51.2%) | 530 (54.1%) | 164 (50.2%) | 118 (50.6%) |  |
| Moderate | 629 (16.9%) | 772 (16.4%) | 330 (14.3%) | 122 (12.5%) | 37 (11.3%) | 23 (9.9%) |  |
| Heavy | 872 (23.4%) | 961 (20.4%) | 404 (17.6%) | 166 (17.0%) | 51 (15.6%) | 51 (21.9%) |  |
| Smoking [N (%)] |  |  |  |  |  |  | <0.001 |
| No | 2205 (59.3%) | 2627 (55.8%) | 1252 (54.4%) | 485 (49.5%) | 162 (49.5%) | 108 (46.4%) |  |
| Yes | 1515 (40.7%) | 2078 (44.2%) | 1049 (45.6%) | 494 (50.5%) | 165 (50.5%) | 125 (53.6%) |  |
| Hypertension [N (%)] |  |  |  |  |  |  | <0.001 |
| No | 2686 (72.2%) | 2784 (59.2%) | 1059 (46.0%) | 347 (35.4%) | 118 (36.1%) | 78 (33.5%) |  |
| Yes | 1034 (27.8%) | 1921 (40.8%) | 1242 (54.0%) | 632 (64.6%) | 209 (63.9%) | 155 (66.5%) |  |
| Diabetes [N (%)] |  |  |  |  |  |  | <0.001 |
| No | 3440 (92.5%) | 4148 (88.2%) | 1883 (81.8%) | 714 (72.9%) | 237 (72.5%) | 155 (66.5%) |  |
| Yes | 280 (7.5%) | 557 (11.8%) | 418 (18.2%) | 265 (27.1%) | 90 (27.5%) | 78 (33.5%) |  |
| CKD [N (%)] |  |  |  |  |  |  | <0.001 |
| No | 3343 (89.9%) | 4006 (85.1%) | 1778 (77.3%) | 691 (70.6%) | 229 (70.0%) | 148 (63.5%) |  |
| Yes | 377 (10.1%) | 699 (14.9%) | 523 (22.7%) | 288 (29.4%) | 98 (30.0%) | 85 (36.5%) |  |
| CKD prognosis risk  [N (%)] |  |  |  |  |  |  | <0.0001 |
| Very high risk | 31 (0.8%) | 76 (1.6%) | 64 (2.8%) | 44 (4.5%) | 17 (5.2%) | 16 (6.9%) |  |
| High risk | 58 (1.6%) | 123 (2.6%) | 114 (5.0%) | 69 (7.0%) | 15 (4.6%) | 16 (6.9%) |  |
| Moderately increased risk | 288 (7.7%) | 500 (10.6%) | 345 (15.0%) | 175 (17.9%) | 66 (20.2%) | 53 (22.7%) |  |
| Low risk | 3343 (89.9%) | 4006 (85.1%) | 1778 (77.3%) | 691 (70.6%) | 229 (70.0%) | 148 (63.5%) |  |
| CKD stages [N (%)] |  |  |  |  |  |  | <0.0001 |
| Stage 1 | 2698 (72.5%) | 2917 (62.0%) | 1222 (53.1%) | 460 (47.0%) | 146 (44.6%) | 116 (49.8%) |  |
| Stage 2 | 863 (23.2%) | 1479 (31.4%) | 828 (36.0%) | 383 (39.1%) | 134 (41.0%) | 87 (37.3%) |  |
| Stage 3 | 144 (3.9%) | 276 (5.9%) | 218 (9.5%) | 124 (12.7%) | 43 (13.1%) | 24 (10.3%) |  |
| Stage 4 | 8 (0.2%) | 23 (0.5%) | 28 (1.2%) | 9 (0.9%) | 3 (0.9%) | 2 (0.9%) |  |
| Stage 5 | 7 (0.2%) | 10 (0.2%) | 5 (0.2%) | 3 (0.3%) | 1 (0.3%) | 4 (1.7%) |  |
| PHQ-9 score [N (%)] |  |  |  |  |  |  | <0.001 |
| <10 | 3518 (94.6%) | 4379 (93.1%) | 2057 (89.4%) | 802 (81.9%) | 267 (81.7%) | 169 (72.5%) |  |
| ≥10 | 202 (5.4%) | 326 (6.9%) | 244 (10.6%) | 177 (18.1%) | 60 (18.3%) | 64 (27.5%) |  |

HbA1c: glycosylated hemoglobin; ALT: glutamic pyruvic transaminase; AST: glutamic oxaloacetic transaminase; TG: triglyceride; TCHOL: total cholesterol; HDL: high-density lipoprotein; LDL: low density lipoprotein; BMI: body mass index; eGFR: estimated glomerular filtration rate; UACR: urinary albumin to creatinine ratio; CKD: chronic kidney disease; PHQ-9**:** item patient health questionnaire-9.

P-values for continuous variables were derived through the application of the Kruskal Wallis rank sum test, while for count variables with theoretical numbers less than 10, P-values were obtained using Fisher's exact probability test.

**Supplement table 2 The results of univariate analysis for eGFR.**

| Variable | eGFR (mL/min/1.73 m^2^)  β (95%CI) | P value |
| --- | --- | --- |
| Age (years) | -0.9 (-0.9, -0.9) | <0.0001*** |
| Sex |  |  |
| Female | 0 |  |
| Male | 0.1 (-0.8, 1.0) | 0.7827 |
| Race |  |  |
| Non-Hispanic white | 0 |  |
| Non-Hispanic black | 11.5 (10.2, 12.7) | <0.0001*** |
| Mexican American | 16.5 (14.7, 18.3) | <0.0001*** |
| Other | 8.9 (7.6, 10.2) | <0.0001*** |
| Fasting blood glucose (mmol/L) | -1.6 (-1.9, -1.2) | <0.0001*** |
| Cr (mmol/L) | -0.4 (-0.5, -0.3) | <0.0001*** |
| UACR (mg/g) | -0.0 (-0.0, -0.0) | <0.0001*** |
| HbA1C (%) | -4.0 (-4.6, -3.4) | <0.0001*** |
| ALT (U/L) | 0.1 (0.0, 0.1) | <0.0001*** |
| AST (U/L) | -0.0 (-0.0, 0.0) | 0.7207 |
| Uric acid (umol/L) | -0.1 (-0.1, -0.1) | <0.0001*** |
| TG (umol/L) | -3.3 (-4.2, -2.5) | <0.0001*** |
| TCHOL (umol/L) | -2.2 (-2.8, -1.6) | <0.0001*** |
| LDL (umol/L) | -2.8 (-4.1, -1.5) | 0.0002*** |
| BMI (kg/m^2^) | -0.1 (-0.1, -0.0) | 0.0391* |
| Nocturnal urination frequency | -3.5 (-4.0, -3.0) | <0.0001*** |
| Drinking (degree) |  |  |
| Never | 0 |  |
| Mild | -2.7 (-4.5, -0.9) | 0.0043*** |
| Moderate | 4.8 (2.8, 6.8) | <0.0001*** |
| Heavy | 10.0 (8.0, 12.0) | <0.0001*** |
| Smoking |  |  |
| No | 0 |  |
| Yes | -1.3 (-2.5, -0.1) | 0.0364** |
| Hypertension |  |  |
| No | 0 |  |
| Yes | -14.8 (-15.8, -13.7) | <0.0001*** |
| Diabetes |  |  |
| No | 0 |  |
| Yes | -12.4 (-13.9, -10.8) | <0.0001*** |
| CKD |  |  |
| No | 0 |  |
| Yes | -22 (-24.7, -21.1) | <0.0001*** |
| CKD prognosis risk |  |  |
| Low risk | 0 |  |
| Moderately increased risk | -14.9 (-16.7, -13.1) | <0.0001*** |
| High risk | -34.4 (-38.2, -30.6) | <0.0001*** |
| Very high risk | -65.7 (-68.4, -62.9) | <0.0001*** |
| CKD stages |  |  |
| Stage 1 | 0 |  |
| Stage 2 | 15.81 (14.22, 17.39) | <0.0001*** |
| Stage 3 | 41.59 (40.26, 42.91) | <0.0001*** |
| Stage 4 | 69.68 (68.47, 70.88) | <0.0001*** |
| Stage 5 | 100.24 (98.98, 101.50) | <0.0001*** |
| PHQ-9 score |  |  |
| <10 | 0 |  |
| ≥10 | 0.9 (-0.8, 2.7) | 0.2835 |

HbA1c: glycosylated hemoglobin; ALT: glutamic pyruvic transaminase; AST: glutamic oxaloacetic transaminase; TG: triglyceride; TCHOL: total cholesterol; HDL: high-density lipoprotein; LDL: low density lipoprotein; BMI: body mass index; eGFR: estimated glomerular filtration rate; UACR: urinary albumin to creatinine ratio; CKD: chronic kidney disease; PHQ-9: item patient health questionnaire-9. *: P<0.05; ***: P<0.001.

**Supplementary table 3 Interaction analysis to assess factors influencing the association between nocturnal urination frequency and eGFR.**

| Variable | β (95% CI) P value | P value | P interaction |
| --- | --- | --- | --- |
| Age |  |  | 0.2902 |
| <60 | -1.1 (-1.4, -0.7) | <0.0001 |  |
| ≥60 | -0.8 (-1.2, -0.3) | 0.0005 |  |
| Sex |  |  | 0.0583 |
| Female | -0.2 (-0.6, 0.1) | 0.1436 |  |
| Male | 0.2 (-0.1, 0.6) | 0.2276 |  |
| Race |  |  | 0.7739 |
| Non-Hispanic white | 0.2 (-0.2, 0.6) | 0.4201 |  |
| Non-Hispanic black | -0.6 (-1.1, -0.1) | 0.0198 |  |
| Mexican American | 0.3 (-0.3, 0.9) | 0.3869 |  |
| Other | 0.1 (-0.4, 0.7) | 0.6132 |  |
| Cr (mmol/L) |  |  | 0.8136 |
| ≤133 | -0.1 (-0.1, -0.0) | 0.0130 |  |
| 134-177 | 0.0 (-0.5, 0.5) | 0.9311 |  |
| 178-450 | 0.1 (-0.7, 0.9) | 0.7940 |  |
| 451-707 | 3.5 (-0.5, 7.5) | 0.0866 |  |
| >707 | - | - |  |
| Fasting blood glucose (mmol/L) |  |  | 0.9261 |
| <7 | 0.0 (-0.3, 0.3) | 0.9396 |  |
| ≥7 | -0.0 (-0.6, 0.6) | 0.9458 |  |
| HbA1C (%) |  |  | 0.3639 |
| <6.5 | 0.1 (-0.2, 0.3) | 0.7003 |  |
| ≥6.5 | -0.3 (-0.9, 0.4) | 0.4154 |  |
| ALT (U/L) |  |  | 0.5600 |
| Low | -0.2 (-0.7, 0.2) | 0.3308 |  |
| Middle | 0.3 (-0.1, 0.7) | 0.1426 |  |
| High | -0.0 (-0.5, 0.4) | 0.8920 |  |
| AST (U/L) |  |  | 0.8472 |
| Low | -0.1 (-0.6, 0.3) | 0.6551 |  |
| Middle | 0.2 (-0.2, 0.7) | 0.3190 |  |
| High | -0.0 (-0.4, 0.4) | 0.9080 |  |
| Uric acid (umol/L) |  |  | 0.8455 |
| Low | 0.0 (-0.4, 0.5) | 0.8565 |  |
| Middle | 0.1 (-0.3, 0.5) | 0.6250 |  |
| High | 0.1 (-0.3, 0.5) | 0.6277 |  |
| TG (umol/L) |  |  | 0.2674 |
| Low | -0.2 (-0.6, 0.3) | 0.4292 |  |
| Middle | 0.2 (-0.3, 0.6) | 0.4352 |  |
| High | 0.2 (-0.3, 0.6) | 0.4222 |  |
| TCHOL (umol/L) |  |  | 0.3635 |
| Low | 0.0 (-0.4, 0.5) | 0.9568 |  |
| Middle | 0.4 (-0.0, 0.8) | 0.0640 |  |
| High | -0.3 (-0.7, 0.2) | 0.2345 |  |
| HDL (umol/L) |  |  | 0.5931 |
| Low | -0.1 (-0.5, 0.4) | 0.8151 |  |
| Middle | 0.1 (-0.3, 0.5) | 0.6820 |  |
| High | 0.1 (-0.3, 0.6) | 0.6098 |  |
| LDL (umol/L) |  |  | 0.9342 |
| Low | -0.0 (-0.5, 0.4) | 0.8848 |  |
| Middle | 0.3 (-0.2, 0.7) | 0.2147 |  |
| High | -0.1 (-0.5, 0.4) | 0.8088 |  |
| BMI (kg/m^2^) |  |  | 0.4781 |
| <18.5 | -3.2 (-5.5, -0.9) | 0.0059 |  |
| 18.5-23.9 | -0.5 (-1.2, 0.1) | 0.0982 |  |
| 24-29.9 | -1.3 (-1.7, -0.8) | <0.0001 |  |
| ≥30 | -0.8 (-1.2, -0.3) | 0.0005 |  |
| Drinking |  |  | 0.0965 |
| Never | -0.3 (-0.9, 0.3) | 0.2865 |  |
| Mild | 0.4 (0.0, 0.7) | 0.0401 |  |
| Moderate | -0.3 (-1.0, 0.3) | 0.3384 |  |
| Heavy | -0.6 (-1.1, -0.0) | 0.0442 |  |
| Smoking |  |  | 0.9233 |
| No | 0 (-0.4,0.3) | 0.8988 |  |
| Yes | 0 (-0.3,0.4) | 0.9911 |  |
| Hypertension |  |  | 0.0497* |
| No | 0.2 (-0.1,0.6) | 0.2065 |  |
| Yes | -0.3 (-0.6,0.1) | 0.1321 |  |
| Diabetes |  |  | 0.2685 |
| No | 0.1 (-0.2, 0.4) | 0.5479 |  |
| Yes | -0.3 (-0.8, 0.3) | 0.3549 |  |
| CKD |  |  | 0.0298* |
| No | -0.1 (-0.2, 0.0) | 0.0873 |  |
| Yes | 0.2 (-0.1, 0.4) | 0.1265 |  |
| CKD prognosis risk |  |  | 0.0005*** |
| Low risk | -0.1 (-0.1 0.0) | 0.0948 |  |
| Moderately increased risk | -0.0 (-0.2, 0.1) | 0.7680 |  |
| High risk | 0.0 (-0.3, 0.3) | 0.9248 |  |
| Very high risk | 0.8 (0.4, 1.1) | <0.0001 |  |
| PHQ-9 score |  |  | 0.5261 |
| <10 | -0.0 (-0.3, 0.3) | 0.9085 |  |
| ≥10 | 0.2 (-0.5, 0.9) | 0.5253 |  |

HbA1c: glycosylated hemoglobin; ALT: glutamic pyruvic transaminase; AST: glutamic oxaloacetic transaminase; TG: triglyceride; TCHOL: total cholesterol; HDL: high-density lipoprotein; LDL: low density lipoprotein; BMI: body mass index; eGFR: estimated glomerular filtration rate; UACR: urinary albumin to creatinine ratio; CKD: chronic kidney disease; PHQ-9**:** item patient health questionnaire-9. Adjusted other variables besides itself and the interactive items related to itself. *: P<0.05; ***: P<0.001.
